# Monitoring of adenovirus serotypes in environmental samples by combined PCR and melting point analyses

**DOI:** 10.1186/1743-422X-10-190

**Published:** 2013-06-10

**Authors:** Nils Marten Hartmann, Melanie Dartscht, Regine Szewzyk, Hans-Christoph Selinka

**Affiliations:** 1 German Federal Environment Agency (UBA, Umweltbundesamt), Division II 1.4 Microbiological Risks, Corrensplatz 1, 14195 Berlin, Germany

**Keywords:** Adenovirus, Environment, Melting point analysis, qPCR, Sewage, Viral indicator, Virus stability

## Abstract

**Background:**

Human adenoviruses are promising candidates for addressing health risks associated with enteric viruses in environmental waters. Relatively harmless but common, these DNA viruses persist within the population and are generally considered extremely stable, remaining infectious in water for long periods of time. Group-specific or single species detection of human adenoviruses in environmental samples is usually based on polymerase chain reaction assays. Simultaneous identification of specific species or serotypes needs additional processing. Here we present a simple molecular approach for the monitoring of serotypic diversity in the human adenovirus populations in contaminated water sites.

**Methods:**

Diversity patterns of human adenoviruses in environmental samples, collected in an outdoor artificial stream and pond simulation system, were analyzed using a closed tube polymerase chain reaction method with subsequent melting point analysis.

**Results:**

Human adenovirus serotype 41 was the most prominent adenovirus serotype detected in environmental water samples, but melting point analyses indicated the presence of additional adenovirus serotypes.

**Conclusions:**

Based on investigations with spiked and environmental samples, a combination of qPCR and melting point analysis was shown to identify adenovirus serotypes in sewage contaminated water.

## Background

Contamination of surface waters by sewage can be a serious hazard to the environment and a potential risk to human health [1,2]. Microbes, viruses and chemical substances may impact animal and plant reproduction as well as egg and larval development [3-5]. Introduction of high nutrient concentrations can induce eutrophication. Furthermore, microbial pollution can affect the stability and composition of microbial communities or ecosystems, depending on the dimension of contamination and the tolerance of the water system [1,6,7]. Moreover, infections by sewage-derived waterborne pathogens may affect the health of both animals and humans alike [8,9]. Studies indicate that viruses often persist for several months in contaminated waters and are detected far longer than indicator bacteria [10-12]. In addition, detection of infectious viruses is more complicated than detection of indicator bacteria, like *Escherichia coli* and intestinal enterococci, both used to detect fecal contaminations in water, and few viral infectious units may cause disease. For instance, 1–10 infectious particles of rotavirus can cause disease in animal or human models, whereas, at similar levels of exposure, the risk of infection with enteropathogenic bacteria is about 10–10.000 times lower than for viruses [12,13]. Reliable indicators for contaminations with human viruses would be important tools to improve and monitor the quality of water bodies, such as bathing waters or drinking water reservoirs. However, selection of a viral indicator is difficult, since human viruses vary in their abundance, virulence, environmental resistance, transport behavior, epidemiological significance, ecology and also in their methods of detection [12,14,15]. The Contaminant Candidate List (CCL), published by the United States Environmental Protection Agency (EPA), lists priority candidates for research in order to facilitate necessary decisions for further regulations. Among the listed viruses are also human adenoviruses [16]. To achieve a higher level of water quality, the World Health Organization (WHO) suggests to perform risk assessments based on the monitoring of one or more representatives of the large group of enteric viruses, e.g. enteroviruses, astroviruses, enteric adenoviruses, orthoreoviruses, rotaviruses, caliciviruses, hepatitis A or E viruses, in addition to bacterial indicators [17]. Viruses that are more readily detected by current available methods are recommended for routine monitoring, which include the members of the enterovirus, adenovirus and orthoreovirus groups. All of these virus taxa comprise many species, serotypes and genotypes, some of them differing strongly with regard to stability and tenacity.

Detection of viruses in environmental samples is usually based on polymerase chain reaction (PCR) and plaque assays. While the latter are time-consuming and intricate, numbers of infectious viruses may be overestimated by PCR based methods as only nucleic acids are detected. PCR based methods may also be subject to inhibitory effects, but are sensitive in general and results can be obtained within few hours [18]. In addition, virus detection by PCR methods can be used for specific monitoring of multiple virus types simultaneously, while plaque assays may completely fail to detect certain virus types, when fast growing competitive strains are present [19,20]. For example human adenovirus [hAdV] serotype 2 is released twice as fast by infected HEK293 cells as the slow growing fecal adenoviruses and may completely lyse a cell layer before human adenovirus serotype 41 will show any visible effect, especially when different amounts of these viruses are present in the same sample [21]. Moreover, adenovirus cultivation is clearly influenced by the cell line used. In particular, using plaque systems, detection of hAdV41 is critical in the presence of species C adenovirus serotypes, while simultaneous detection and quantification of both serotypes is no problem using multiplex quantitative real time PCR (qPCR) [22,23]. However, infectivity cannot be demonstrated by direct PCR measurements alone, but by application of cell culture-based methods, like plaque assays or integrated cell culture PCR (ICC/qPCR) [24]. As long as no better approaches are available and affordable, monitoring should ideally be performed by combining PCR and cell culture-based assays [22].

Most methods for virus detection and typing were designed for testing of clinical samples, and only few of them can be applied to environmental samples without modifications and additional processing. While many virus methods are either suitable for detection, quantification or typing, melting point analysis (MPA) is an inexpensive and fast alternative, which can be directly connected to qPCR amplification without further processing and additional equipment. Since even single nucleotide exchanges lead to shifts in the melting points of specific sequences, the technique is often used for mutation scanning in clinical samples [25,26]. However, so far, this method has not been applied to environmental samples for characterization of viruses. Here, we demonstrate the use of PCR product melting points for heterogeneity analyses and its suitability for direct identification of human adenovirus serotypes in sewage-contaminated samples.

## Results

**Figure 1:**
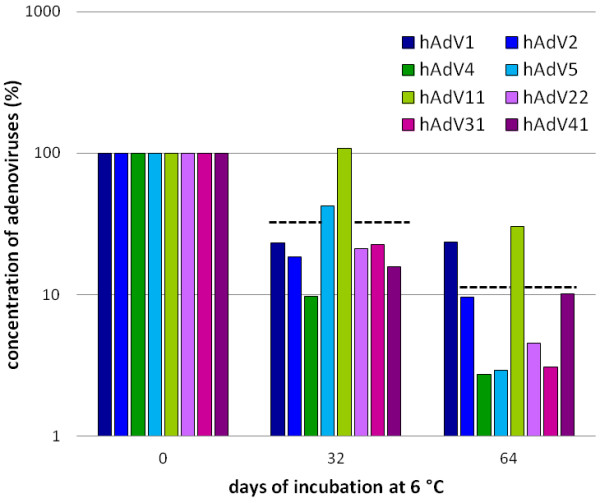
**Stabilities of human adenovirus serotypes in processed water at 6°C.** Concentrations were determined by qPCR in four-week intervals. Decreases of virus copy numbers at 6°C did not exceed 1.5 log_10_ units for any tested serotype. Dashed lines represent calculated mean concentrations.

**Figure 2:**
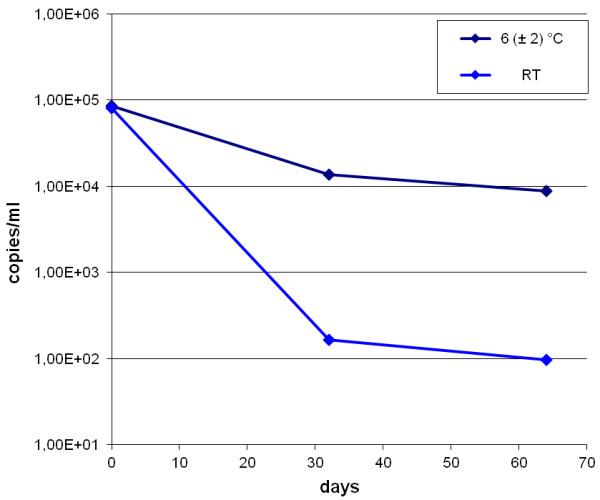
**Decrease of human adenovirus serotype 41 in processed water at distinct temperatures over time.** The decrease of virus copy numbers at room temperature exceeded the decrease at 6°C by about 2 log_10_ units.

Serotypes of all known human adenovirus species, except for the single serotype in species G, were tested for their stability in processed water in order to investigate their potential use as viral indicators. Human adenovirus serotypes 1, 2 and 5 (species C), 4 (species E), 11 (species B), 22 (species D), 31 (species A) and 41 (species F) were investigated. Concentrations of all examined serotypes did not decrease for more than 1.5 log_10_ steps at 6 (± 2)°C within eight weeks (Figure 1). While some viruses decreased stronger during the early incubation phase (serotypes 1, 2, 4 and 41), others showed a higher logarithmic decrease during later times (serotypes 5, 11 and 31), whereas hAdV22 decreased constantly over time. The mean reduction in copy numbers of all tested adenoviruses was in the range of about 0.5 log in 32 days and about 1.0 log in 64 days. hAdV41 displayed a decrease in copy numbers of 1.0 log_10_ unit at 6°C and 3.0 log_10_ units at room temperature (Figure 2). Regarding virus stability, the results demonstrate, that all of the tested serotypes would potentially qualify for biodiversity analyses or could be used as indicators for contaminations with human viruses derived from municipal sewage.

For melting point analyses following qPCR amplifications, the primer pair AdF/AdR, designed by Hernroth *et al.* (2002), was used [27]. Distinct melting points (T_m_) were identified for several adenovirus serotypes. Melting points and relative shifts largely confirmed theoretical calculations using sequences provided by NCBI, amended by the primer sequences, and the OligoCalc program [28]. Since degenerated primers were used, melting points of serotype-specific amplification products are listed in temperature ranges (Table 1).

**Table 1:** Comparison of calculated and measured melting points

**Serotype**	**Species**	**Calculated T**_**m**_	**Measured T**_**m**_
hAdV11	B	81.03 to 81.82°C	81,10 ± 0.19°C
hAdV4	E	81.37 to 82.14°C	81,69 ± 0.30°C
hAdV31	A	81.66 to 82.41°C	82,11 ± 0.23°C
hAdV22	D	83.08 to 83.32°C	83,28 ± 0.19°C
hAdV41	F	83.47 to 84.16°C	83,28 ± 0.27°C
hAdV40	F	83.57 to 84.25°C	80,38 ± 1.23°C
hAdV1	C	83.57 to 84.25°C	83,78 ± 0.06°C
hAdV2	C	83.57 to 84.25°C	83,26 ± 0.48°C
hAdV5	C	83.57 to 84.25°C	83,86 ± 0.19°C

**Figure 3:**
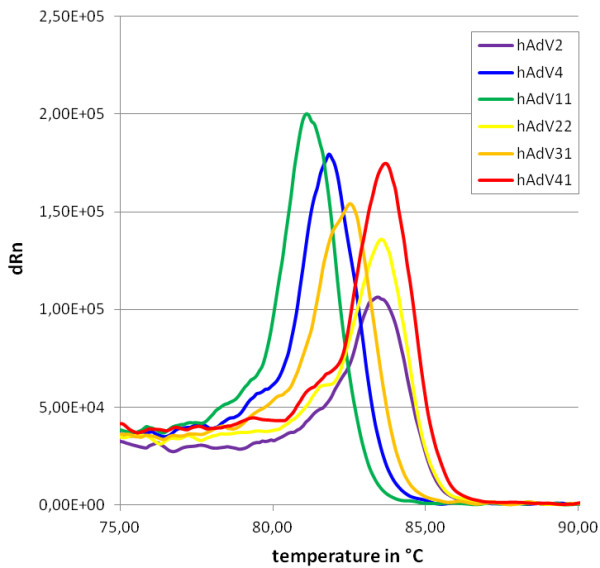
**Melting curves of different human adenovirus serotypes.** Melting curves were determined following qPCR amplification. Differences of only 1 to 3 bp in the amplified regions led to significant differences in melting temperatures.

Typical melting curves for several human adenovirus serotypes are shown in Figure 3. Peak positions of the type-specific adenovirus melting curves were mostly stable and standard deviations were generally low (Table 1). Serotypes hAdV2, hAdV22 and hAdV41 showed overlying melting curves with maxima too close for discrimination in any serotype mixed sample. Similar results were obtained for hAdV1 and hAdV5, belonging to the same adenovirus species as hAdV2 (data not shown). However, several adenovirus serotypes displayed clear distinguishable peak positions.

**Figure 4:**
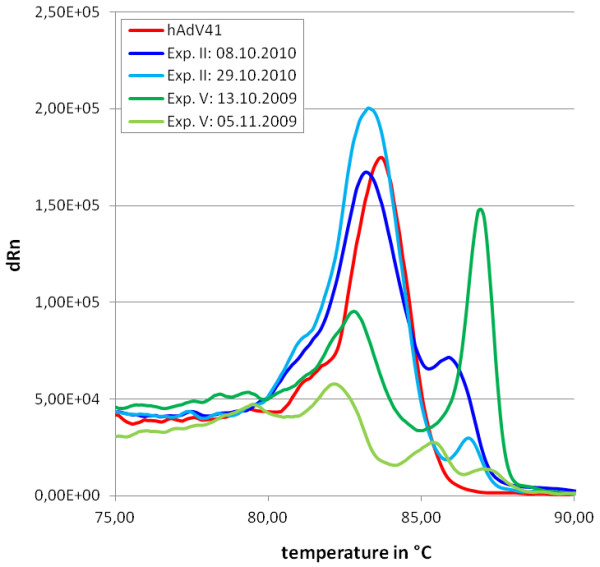
**Melting curves of environmental water samples.** The melting curves of the human adenovirus serotype 41 reference and four environmental samples are shown. Each curve represents the mean value of two parallels. Equivalent results were obtained during several runs.

**Figure 5:**
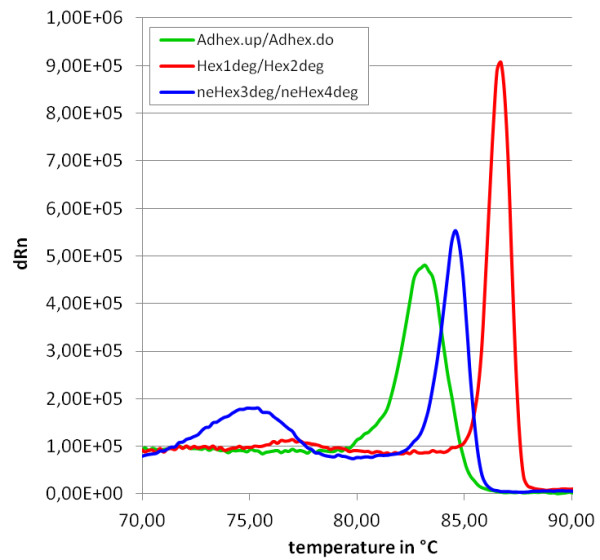
**Melting curves of an adenovirus plasmid standard using different sets of primers.** The plasmid contained an insert of the human adenovirus serotype 41 hexon gene. Three sets of primers were used for amplification of the insert, producing amplicons of different lengths. Melting temperatures correlated with the lengths of amplicons. The small blue peak at around 75°C corresponds to the primer peak of the neHex3deg/neHex4deg system.

In a second set of experiments, melting point analysis was applied to environmental water samples. These samples derived from tenacity experiments performed in an artificial stream and pond simulation system with sewage-contaminated water containing human adenoviruses. Samples were drawn in the beginning and at the end of experiments, spanning a time interval of several weeks. Conspicuous in some graphs were pronounced shoulders or multiple peaks, putatively indicating serotype heterogeneity (Figure 4). In several cases peak intensities declined dramatically over time, as exemplarily shown for experiment V (green lines). The peak positioned at about 84°C, most often the highest or main peak, was always overlapping with the reference peak (fecal adenovirus serotype 41). These results were also confirmed by restriction fragment analyses, and, for some samples (exp. II), by direct sequencing (data not shown). In contrast, high temperature peaks (peaks exceeding 85°C) could not be assigned to any reference virus used for melting point analyses in our experiments. Since the applied primer system was originally designed for group-specific adenovirus detection, primers contained degenerated base positions. Therefore, the specificity of the primers was analyzed for all known human adenovirus serotypes (56 serotypes) and several zoopathogenic mastadenovirus species. However, none of the predicted melting points surpassed 85°C. Table 2 lists the number of nucleotide differences of primer and target sequences. Only the reference serotypes as well as hAdV3 (species B, maximal nucleotide mismatch number), hAdV52 (species G) and certain animal serotypes are displayed. The number of mismatches was identical for all serotypes of the species C and D, respectively. hAdVE and hAdVG consist of only one known serotype (serotype 4 and serotype 52, respectively). Significant nucleotide matches with non-adenovirus sequences were not predicted by blastn, and highest alignments were always below 90%. The primer pair AdF/AdR is usually used in combination with the TaqMan probe ACDEF for group-specific adenovirus qPCR [27]. Therefore, both the differences in probe and target sequences were determined (Table 2). Although primer alignments showed higher mismatch numbers for most investigated animal adenoviruses than for human adenoviruses, most of the mismatches concerning the reverse primer AdR are located at the 5'-prime end, the end which is not extended during polymerization. However, during qPCR the number of mismatches will most likely prevent probe association in most cases. In our melting point analyses SYBR green was used and detection of non-human adenoviruses cannot be excluded. Therefore, other primer systems were investigated for their specificity and applicability in melting point analyses, namely the primer pairs Hex1deg/Hex2deg and neHex3deg/neHex4deg (Figure 5). These primer pairs were originally designed for nested PCR based detections of human adenoviruses [29]. While 69 base pair (bp) amplicons were produced using the AdF/AdR primer pair, 173 bp amplicons were produced using the neHex3deg/neHex4deg primer pair and 301 bp amplicons by using the Hex1deg/Hex2deg primer pair [27,29]. Sequence differences between related serotypes where expected to be higher in longer amplicon sequences and thus expected to allow better melting point discriminations. Analysis of primer specificities using the neHex3deg/neHex4deg primer pair instead of the AdF/AdR primer pair revealed lower affinities for animal adenovirus sequences than for human adenovirus sequences. Differences of animal and human adenovirus sequences using the Hex1deg/Hex2deg primer pair were comparable to differences using the AdF/AdR primer pair, with generally slightly reduced specificities using the latter primer pair. QPCR analyses yielded comparable results for all three primer pairs, using the pAdV41 standard as target. Although higher melting temperatures were achieved with longer primer sequences, amplification efficiencies for the different serotypes were best using the AdF/AdR primer system (data not shown). The choice of appropriate primer pairs is therefore critical for the monitoring of virus serotypes in environmental water samples.

**Table 2:** Nucleotide mismatches between primer, probe and target sequences

**Serotype or species**^**a**^	**Fw primer**^**b**^	**Rv primer**^**b**^	**Probe**^**b**^
hAdV31 [A]	0	0	3
hAdV3 [B]	0	0	4
hAdV11 [B]	0	0	3
hAdV2 [C]	0	0	1
hAdV22 [D]	0	0	0
hAdV4 [E]	0	0	2
hAdV40 [F]	0	0	0
hAdV41 [F]	0	0	1
hAdV52 [G]	0	0	2
bAdVA	1	3	6
cAdV1	2	3	7
cAdV2	2	3	7
fAdV	0	0	2
mAdV2	1	2	5
oAdVA	0	3	7
pAdVC	1	3	3

^a^ Human adenovirus species are listed in brackets. For abbreviations and accession numbers, see Methods section.

^b^ Forward primer, reverse primer and probe, designed according to Hernroth *et al.* (2002).

## Discussion

Temperature is an important trigger to abiotic virus destruction. Therefore stability analyses with selected adenovirus serotypes were performed. The majority of the examined serotypes displayed similar innate stabilities at 6°C. Virus stabilities showed some variation within the range of 1.0 log_10_ steps after the incubation of 64 days. All serotypes could still be detected after this time period and copy numbers of specific serotypes did never decrease for more than 1.5 log_10_ steps. Under environmental conditions, where temperatures and other parameters vary over time, stronger deviations may be observed. Species F adenovirus serotypes (hAdV40 and hAdV41) show high prevalence in environmental samples, but the results indicate, that other serotypes may be stable enough to allow diversity analyses up to weeks after contamination.

For such investigations, melting point analysis was adapted to adenovirus serotyping. The analyses of AdF/AdR amplification products confirmed the calculated melting points with high accuracy. Only serotype 40 was not within the range of prediction. This may be due to genotypic variations and characterization of this specimen is ongoing. The presented investigations using adenoviruses from virus cultures suggest, that discrimination of serotypes is possible when corresponding melting points differ by more than 0.3°C. Although only a restricted number of different serotypes could be discriminated by the primer system used, the results were consistent and seem to allow a fast classification of specimens in samples with defined matrices. Samples with a small number of serotypes may even be judged on the basis of melting curve analyses alone, using an appropriate set of melting point reference viruses. Several research groups have recently demonstrated that discrimination of norovirus, influenzavirus and fowl adenovirus species or serotypes by melting point analysis is possible [30-32]. None of these groups analyzed environmental samples, but their investigations and our current findings with human adenoviruses suggest that serotype discrimination of other virus taxa by melting point analysis is also possible in environmental samples.

Heterogeneity analyses of sewage-derived samples revealed multiple peaks or pronounced shoulders in several, but not all samples. Unambiguous sequences were obtained by direct sequencing from samples with single peak melting curves and samples with melting curves showing small shoulders, indicating that shoulders might be of artificial nature, for instance a result of insufficient purity or interfering components [33]. However, failure of heterogeneity detection by direct sequencing might simply result from concentration differences of distinct sequence products in the samples. In contrast, samples with multiple peaks could not be sequenced without cloning, suggesting heterogeneity. In complex samples, peaks may fuse and broaden, hindering correlation attempts. Therefore, we assume that, in contrast to the in vitro experiments with purified adenovirus serotypes, melting point differences of at least 1–1.5°C are required for the analysis of serotype heterogeneity in environmental samples.

Peaks exceeding 85°C could not be assigned to any accessible sequences of human or animal adenoviruses. Recent studies revealed tremendous numbers of different and as yet mostly uncharacterized species from almost every sampling site, that will employ molecular biologists as well as ecologists, taxonomists, biochemists and others for years to come [34-36]. Such high genetic diversity increases the potential risk of false positive results generated by unspecific amplification and insufficient primer design. Even if primer specificity is high, detection of new kinds of human or animal pathogenic adenoviruses is not unlikely. New animal and human adenovirus serotypes are described frequently and only limited knowledge seems to be available concerning the ecology and interspecies transmission of many serotypes [37]. Although adenoviruses were assumed to be host specific, new findings suggest that for example the feline or several primate adenoviruses are not [38-40]. It is unknown how many adenoviruses can cause zoonosis or anthroponosis, and some of them may also be amplified by the primer system used. Melting points of sequences directly depend on base composition and sequence length. The highest peak obtained from our environmental samples, measured at 86.43 ± 0.24°C using the AdF/AdR primer pair might belong to an unknown human or animal adenovirus with significant sequence differences to the investigated serotypes.

Revealing of unknown sequences in anthropogenic sewage by a simple method like melting point analysis underlines the urgent need for basic research in the field of viral biodiversity and virus ecology in particular, and genetic diversity in general. Samples hinting at unknown adenoviruses by means of melting point analyses are currently undergoing further investigation in our working group. The combined method of qPCR and melting point analyses could be used to monitor the current epidemiological status of certain virus serotypes in contaminated environmental water samples. For this assay viral nucleic acids were concentrated from 10 L samples, but smaller sample volumes could be suitable as well, provided that virus titers are high enough. Methods with better recovery rates will also improve the possibilities to use serotype analysis approaches.

In contrast to results using the adenovirus AdF/AdR primers, efficiencies of serotype detection varied using the Hex1deg/Hex2deg and neHex3deg/neHex4deg primer pairs. Whereas some serotypes were amplified equally well using different primer systems, others displayed variation. Longer sequences did not allow a better discrimination, although the peaks were generally sharper. In contrast, shorter sequences usually produced broader peaks. These results indicate, that careful selection of the primer system used for the PCR preceding the melting point analyses is crucial. Although exact serotyping will always need confirmation by sequencing of the amplicons, melting point analyses can provide fast information regarding the heterogeneity within a specific sample. It therefore may be a helpful tool to decide, whether further, probably costly and time-consuming investigations with regard to virus diversity, are justified.

Based on the results of melting point analyses and stability investigations, fecal adenovirus serotype 41 clearly qualified as an indicator for sewage pollution. Ad41 showed an innate stability comparable to other human pathogenic adenoviruses and was detected in all sewage derived samples. It therefore may be used for monitoring of adenovirus elimination during water treatment.

To our best knowledge, this study is the first application of melting point analyses on viruses in environmental water samples. Our results demonstrate, that several human adenovirus serotypes can be distinguished on the basis of their melting behavior. Melting point analyses of environmental samples are inexpensive, fast and accomplishable in a closed tube system, subsequent to PCR.

The described application of this method on sewage-contaminated water samples revealed its suitability for environmental samples. Although it is not required for routine monitoring, the method described in this research could be useful for epidemiological investigations.

## Conclusion

Although human adenoviruses type 40 and 41 are the dominating serotypes in environmental water samples, other adenovirus serotypes may be present. Water samples obtained from processed groundwater which was artificially contaminated with 1% sewage was used to prove the principle that human adenovirus serotype diversity can be monitored using a combination of group-specific qPCR and melting curve analysis. Such melting curve analyses on PCR amplification products of environmental samples may be used as a first screening step on samples intended for further epidemiologic serotype analyses.

## Methods

### Viruses

Human adenovirus serotypes 1 (species C), 4 (species E), 11 (species B), 22 (species D), 31 (species A) and 40 (species F) were provided as culture supernatants by the German National Reference Laboratory for Adenoviruses, Hannover Medical School, Germany. Human adenoviruses serotypes 2 and 5 (species C) and 41 (species F) were purchased from the American type culture collection (ATCC) and cultivated in HEK 293 cells.

### Virus stability investigations

For the investigation of virus stabilities, various adenovirus serotypes were diluted in processed water (ground water after iron/manganese removal) obtained from the UBA water facility in Berlin-Marienfelde. A total volume of 20 ml was spiked with either one or multiple human adenovirus serotypes (total concentrations of 10^5^ viruses). The volume of 20 ml was chosen to avoid further concentration steps for virus recovery. Virus stability was investigated over a period of eight weeks and samples were collected every four weeks. All approaches were protected from UV light and kept at either cool (6 ± 2°C) or ambient temperatures (room temperature, 20-25°C), without stirring. These temperatures were chosen as representatives for cool and warm environmental conditions. 1 ml aliquots were analyzed after nucleic acid extraction by TaqMan adenovirus consensus qPCR using the AdF/AdR primer system [27].

### Environmental samples

Environmental water samples containing naturally circulating human adenoviruses were drawn during four long-time experiments, performed in fall 2009, spring and autumn 2010, and fall 2011, using the artificial stream and pond system (FSA) of the German Federal Environment Agency (UBA) in Berlin. Processed water (ground water after biological iron/manganese removal), was spiked to a final concentration of 1-5% (v/v) sewage using primary effluent. For the present serotype analysis study, 10 L samples were collected at the start of these experiments and after a time-span of about one month. Sample concentration by glass wool filtration and flocculation was performed as described by Wyn-Jones *et al.* (2010) [41].

### Nucleic acid extraction

Nucleic acid extraction was performed using the NucliSens Magnetic Extraction Kit (Biomérieux) according to the manufacturer’s instructions. Briefly, samples were lysed in 2 volumes of lysis buffer for 10 min after mixing. 50 μl of magnetic bead suspension were added, mixed and incubated for another 10 minutes. Magnetic beads were collected by a magnetic holder (Novagen) and washed with three different buffers, according to the manufacturer’s instructions. For elution a total of 100 μl elution buffer was used in two succeeding steps, but collected in one tube. Elution was carried out in a thermoshaker (Eppendorf) for 5 min at 60°C and 14000 rpm (each step).

### Combination of quantitative real time PCR and melting point analyses

For quantitative real time PCR undiluted or 1:10 diluted nucleic acid samples were measured against a standard curve of plasmid DNA containing an insert from the hexon gene sequence of adenovirus serotype 41 (pAdV41). The standard curve was performed using concentrations from 10^1^ to 10^6^ copies in 3 parallels, respectively. 10 μl of diethylpyrocarbonate (DEPC)-treated, deionized water served as non-template control, while three parallels of 5 × 10^2^ copies of standard were used as positive controls. Reference virus concentrations were about 10^3^ copies per sample. Each sample and standard, as well as positive and negative controls used during adenovirus investigations contained 12.5 μl SYBR-Green Mastermix (Applied Biosystems), 0.9 μM forward primer and reverse primer, respectively, and 0.5 μl of DEPC treated and sterile water. Of each sample at least two parallels were measured. The primer systems used in this study are based on consensus sequences within the hexon gene region of the adenoviral genome. This conserved gene region is similar in all human and many mammalian adenovirus serotypes and encodes for the capsid hexon protein. Three different sets of forward and reverse primers were used during these investigations (see Results for further information): AdF (5'-CWT ACA TGC ACA TCK CSG G-3') and AdR (5'-CRC GGG CRA AYT GCA CCA G-3'), Hex1deg (5'-GCC SCA RTG GKC WTA CAT GCA CAT C-3') and Hex2deg (5'-CAG CAC SCC ICG RAT GTC AAA-3') and neHex3deg (5'-GCC CGY GCM ACI GAI ACS TAC TTC-3') and neHex4deg (5'- CCY ACR GCC AGI GTR WAI CGM RCY AAC TA-3'). Primer systems were designed by Henroth *et al.* 2002 (AdF/AdR) and Allard *et al.* 2001 (Hex1deg/Hex2deg and neHex3deg/neHex4deg) [27,29]. Total volume was always 25 μl. Standards and primers were solved in DEPC-treated, deionized water. MicroAmp Optical 96-Well Reaction Plates (Applied Biosystems) were used during measurement. The qPCR protocol comprised an initial hold at 95°C for 10 min (hot start), followed by 45 cycles of a 2-step PCR protocol with a 15 sec denaturation phase at 95°C and a 1 min annealing/elongation phase at 60°C. All qPCR experiments were performed using the Applied Biosystem 7500 RT-PCR Fast system. System software version 2.0.5 was used for analyses.

DNA melting temperatures were measured directly after qPCR amplification according to the Applied Biosystem melting curve protocol. In short, after qPCR amplification the samples were heated to 95°C for 15 sec, cooled down to 60°C (60 sec) and subsequently heated to 95°C (30 sec) with a ramp rate of 0.5%. Finally, the samples were cooled down to 60°C (15 sec). T_m_ values (mean values of several measurements) were taken from the first deviations of the melting curves. Without further software, export of the melting curves is possible, but first deviations have to be calculated manually. Our graphical data represent mean values of 2–5 melting curves. The analysis of 1:10 diluted samples derived from FSA experiments was followed by direct sequencing from the product mixture, performed by Dr. Martin Meixner GmbH, Berlin.

### Sequence alignments and melting point calculations

The following human adenovirus serotypes were used for sequence alignments, NCBI GenBank or RefSeq accession numbers are listed in square brackets: hAdVA serotypes: 12 [X73487.1], 18 [DQ149610.1] and 31 [DQ149611.1]; hAdVB serotypes: 3 [DQ086466.1], 7 [AF515814.1], 11 [AB330092.1], 14 [DQ149612.1], 16 [X74662.1], 21 [AY008279.1], 34 [AB052911.1], 35 [AB052912.2], 50 [DQ149643.1] and 55 [FJ643676.1]; hAdVC serotypes: 1 [AF534906.1], 2 [J01917.1], 5 [AC_000008.1] and 6 [DQ149613.1]; hAdVD serotypes: 8 [DQ149614.1], 9 [AJ854486.1], 10 [DQ149615.1], 13 [DQ149616.1], 15 [DQ149617.1], 17 [AF108105.1], 19 [DQ149618.1], 20 [DQ149619.1], 22 [DQ149620.1], 23 [DQ149621.1], 24 [DQ149622.1], 25 [DQ149623.1], 26 [DQ149624.1], 27 [DQ149625.1], 28 [DQ149626.1], 29 [DQ149627.1], 30 [DQ149628.1], 32 [DQ149629.1], 33 [DQ149630.1], 36 [DQ149631.1], 37 [DQ149632.1], 38 [DQ149633.1], 39 [DQ149634.1], 42 [DQ149635.1], 43 [DQ149636.1], 44 [DQ149637.1], 45 [DQ149638.1], 46 [AY875648.1], 47 [DQ149640.1], 48 [EF153473.1], 49 [DQ393829.1], 51 [DQ149642.1], 53 [FJ169625.1], 54 [AB448770.2] and 56 [HM770721.2]; hAdVE serotypes: 4 [AY487947.1]; hAdVF serotypes: 40 [AB330121.1] and 41 [AB330122.1]; hAdVG serotypes: 52 [DQ923122.2]. Non-human adenoviruses used in the analysis were the bovine adenovirus A (bAdVA) [NC_006324.1], canine adenovirus serotypes 1 (cAdV1) [AC_000003.1] and 2 (cAdV2) [DQ839392.1], the feline adenovirus (fAdV) [AY512566.1], the murine adenovirus serotype 2 (mAdV2) [NC_014899.1], the ovine adenovirus A (oAdVA) [NC_002513.1] and the porcine adenovirus C (pAdVC) [NC_002702.1]. Melting point calculations were using the OligoCalc program with parameters of 900 nm primer and 50 mM salt concentrations [28].

## Competing interests

The authors declare that they have no competing interests.

## Authors’ contributions

NMH conceived this study, designed and coordinated the experiments, participated in all experiments performed, analyzed and interpreted the data and wrote the manuscript. MD performed several important experiments, including sample preparation and adenovirus serotyping from sewage-derived samples by restriction fragment analyses. HCS co-designed the study, participated in data analysis and revision of the manuscript. RS participated in interpreting the data and revised the manuscript. All authors read and approved the final manuscript.

## References

[B1] Longe EO Ogundipe AO Assessment of wastewater discharge impact from a sewage treatment plant on lagoon later, Lagos, Nigeria Res J Appl Sci Eng Technol 2010 2 3 274 282

[B2] Hrudey SE Hrudey EJ Published case studies of waterborne disease outbreaks – evidence of a recurrent threat Water Environ Res 2007 79 3 233 245 10.2175/106143006X95483 17469655

[B3] Coelho SM Rijstenbil JW Brown MT Impacts of anthropogenic stresses on the early development stages of seaweeds J Aquat Ecosyst Stress Recovery 2000 7 4 317 333 10.1023/A:1009916129009

[B4] Jobling S Nolan M Tyler CR Brighty G Sumpter JP Widespread sexual disruption in wild fish Environ Sci Technol 1998 32 17 2498 2506 10.1021/es9710870

[B5] Calabrese A How some pollutants affect embryos and larvae of american oyster and hard-shell clam Mar Fish Rev 1972 34 11–12 66 77

[B6] Khan FA Ansari AA Eutrophication: An ecological vision Bot Rev 2005 71 4 449 482 10.1663/0006-8101(2005)071[0449:EAEV]2.0.CO;2

[B7] Smith SV Neilson BJ, Cronin LE Responses of Kaneohe Bay, Hawaii, to relaxation of sewage stress Estuaries and Nutrients 1981 Clifton, New Jersey: Humana Press Inc 391 410

[B8] Appelbee AJ Thompson RCA Measures LM Olson ME Giardia and Cryptosporidium in harp and hooded seals from the Gulf of St. Lawrence, Canada Vet Parasit 2010 173 1–2 19 23 10.1016/j.vetpar.2010.06.001 20594649

[B9] WHO Health Based Monitoring of Recreational Waters: The Feasibility of a New Approach (the ‘Annapolis Protocol’) 1999 Geneva: World Health Organisation

[B10] Enriquez CE Hurst CJ Gerba CP Survival of the enteric adenoviruses 40 and 41 in tap, sea, and waste water Water Res 1995 29 11 2548 2553 10.1016/0043-1354(95)00070-2

[B11] Medema GJ Shaw S Waite M Snozzi M Morreau A Grabow W Dufour A, Snozzi M, Köster W, Bartram J, Ronchi E, Fewtrell L Catchment characterisation and source water quality Assessing microbial safety of drinking water: Improving approaches and methods 2003 London, UK: IWA Publishing, on behalf of the World Health Organization and the Organisation for Economic Co-operation and Development 111 158

[B12] Bosch A Human enteric viruses in the water environment: a minireview Internatl Microbiol 1998 1 3 191 196 10943359

[B13] Leclerc H Schwartzbrod L Dei-Cas E Cloete TE, Rose J, Nel LH, Ford T Microbial agents associated with waterborne diseases Microbial waterborne pathogens 2004 London, UK: IWA Publishing London 1 54

[B14] Ashbolt NJ Grabow WOK Snozzi M Fewtrell L, Barthram J Indicators of microbial water quality Water quality: Guidelines, standards and health 2001 London, UK: IWA Publishing 289 316

[B15] Figueras MJ Borrego JJ New perspectives in monitoring drinking water microbial quality Int J Environ Res Public Health 2010 7 12 4179 4202 10.3390/ijerph7124179 21318002 PMC3037048

[B16] US EPA Drinking water contaminant list 3 – Final Fed Reg 2009 74 194 51850 51862

[B17] WHO Guidelines for Drinking-water Quality. Volume 1 2008 3 Geneva: World Health Organization

[B18] Fong T-T Lipp EK Enteric viruses of humans and animals in aquatic environments: Health risks, detection, and potential water quality assessment tools Microbiol Mol Biol Rev 2005 69 2 357 371 10.1128/MMBR.69.2.357-371.2005 15944460 PMC1197419

[B19] Beuret C Simultaneous detection of enteric viruses by multiplex real-time RT-PCR J Virol Meth 2004 11 1 1 8 10.1016/j.jviromet.2003.09.005 14656455

[B20] Aslan A Xagoraraki I Simmons FJ Rose JB Dorevitch S Occurrence of adenovirus and other enteric viruses in limited-contact freshwater recreational areas and bathing waters J Appl Microbiol 2011 111 5 1250 1261 10.1111/j.1365-2672.2011.05130.x 21854513

[B21] Siqueira-Silva J Perez Yeda F Favier A-L Mezin P Silva ML Médici Barrella K Mehnert DU Fender P Hársi CM Infection kinetics of human adenovirus serotype 41 in HEK 293 cells Mem Inst Oswaldo Cruz 2009 104 5 736 744 10.1590/S0074-02762009000500013 19820835

[B22] Jiang SC Han J He J-W Chu W Evaluation of four cell lines for assay of infectious adenoviruses in water samples J Water Health 2009 7 4 650 656 10.2166/wh.2009.088 19590132

[B23] Xu W McDonough MC Erdman DD Species-specific identification of human adenoviruses by a multiplex PCR assay J Clin Microbiol 2000 38 11 4114 4120 11060077 10.1128/jcm.38.11.4114-4120.2000 PMC87550

[B24] Balkin HB Margolin AB Detection of poliovirus by ICC/qPCR in concentrated water samples has a greater sensitivity and is less costly using BGM cells in suspension as compared to monolaysers Virology J 2010 7 282 285 10.1186/1743-422X-7-282 20974002 PMC2978159

[B25] Murugesan G Hsi ED Development of a clinical assay for JAK2 V617F genotyping in chronic myeloproliferative disorders Pathol Res 2006 1 4 10.1309/TK0X-L917-XK2V-LRPQ 16627272

[B26] Reed GH Wittwer CT Sensitivity and specificity of single-nucleotide polymorphism scanning by high-resolution melting analysis Clin Chem 2004 50 10 1748 1754 10.1373/clinchem.2003.029751 15308590

[B27] Hernroth BE Conden-Hansson A Rehnstam-Holm A Girones R Allard AK Environmental factors influencing human viral pathogens and their potential indicator organisms in the blue mussel, *Mytilu eduli*: the first Scandinavian report Appl Environ Microbiol 2002 68 9 4523 4533 10.1128/AEM.68.9.4523-4533.2002 12200309 PMC124092

[B28] OligoCalc Oligonucleotide properties calculator[http://www.basic.northwestern.edu/biotools/oligocalc.html]

[B29] Allard A Albinsson B Wadell G Rapid typing of human adenoviruses by a general PCR combined with restriction endonuclease analysis J Clin Microbiol 2001 39 2 498 505 10.1128/JCM.39.2.498-505.2001 11158096 PMC87765

[B30] Scipioni A Mauroy A Ziant D Saegerman C Thiry E A SYBR green RT-PCR assay in single tube to detect human and bovine noroviruses and control for inhibition J Virol Meth 2008 5 94 1 8 10.1186/1743-422X-5-94 PMC2546391 18702817

[B31] Lin J-H Tseng C-P Chen Y-J Lin C-Y Chang S-S Wu H-S Cheng J-C Rapid differentiation of influenza A virus subtypes and genetic screening for virus variants by high-resolution melting analysis J Clin Microbiol 2008 46 3 1090 1097 10.1128/JCM.02015-07 18174299 PMC2268327

[B32] Steer PA Kirkpatrick NC O’Rourke D Noormohammadi AH Classification of fowl adenovirus serotypes using high resolution melting curve analysis of the hexon gene region J Clin Microbiol 2009 47 2 311 321 10.1128/JCM.01567-08 19036935 PMC2643661

[B33] D’haene B Hellemans J The importance of quality control during qPCR data analysis Int Drug Discov 2010 18 24

[B34] McInerney JO Mullarkey M Wernecke ME Powell R Bacteria and Archaea: Molecular techniques reveal astonishing diversity Biodiversity 2002 3 2 3 10 10.1080/14888386.2002.9712571

[B35] Breitbart M Thompson LR Suttle CA Sullivan MB Exploring the vast diversity of marine viruses Oceanography 2007 20 2 135 139 10.5670/oceanog.2007.58

[B36] Cantalupo PG Calgua B Zhao G Hundesa A Wier AD Katz JP Grabe M Hendrix RW Girones R Wang D Pipas JM Raw sewage harbors diverse viral populations mBio 2011 2 5 e00180 11 21972239 10.1128/mBio.00180-11 PMC3187576

[B37] Maluquer de Motes C Hundesa A Almeida FC Bofill-Mas S Girones R Isolation of a novel monkey adenovirus reveals a new phylogenetic clade in the evolutionary history of simian adenoviruses Virology J 2011 8 125 132 10.1186/1743-422X-8-125 21414228 PMC3068977

[B38] Phan TG Shimizu H Nishimura S Okitsu S Maneekarn N Ushijima H Human adenovirus type 1 related to feline adenovirus: evidence of interspecies transmission Clin Lab 2006 52 9–10 515 518 17078479

[B39] Chen EC Yagi S Kelly KR Mendoza SP Maninger N Rosenthal A Spinner A Bales KL Schnurr DP Lerche NW Chiu CY Cross-species transmission of a novel adenovirus associated with a fulminant pneumonia outbreak in a New World Monkey colony PLOS Pathog 2011 7 7 e1002155 10.1371/journal.ppat.1002155 21779173 PMC3136464

[B40] Wevers D Metzger S Babweteera F Bieberbach M Boesch C Cameron K Couacy-Hymann E Cranfield M Gray M Harris LA Head J Jeffery K Knauf S Lankester F Leendertz SAJ Lonsdorf E Mugisha L Nitsche A Reed P Robbins M Travis DA Zommers Z Leendertz FH Ehlers B Novel adenoviruses in wild primates: a high level of genetic diversity and evidence of zoonotic transmissions J Virol 2001 85 20 10774 10784 21835802 10.1128/JVI.00810-11 PMC3187507

[B41] Wyn-Jones AP Carducci A Cook N D´Agostino MD Divizia M Fleischer J Gantzer C Gawler A Girones R Höller C de Roda Husman AM Kay D Kozyra I López-Pila J Muscillo M São José Nascimento M Papageorgiou G Rutjes S Sellwood J Szewzyk R Wyer M Surveillance of adenoviruses and noroviruses in European recreational waters Water Res 2011 45 3 1025 1038 10.1016/j.watres.2010.10.015 21093010 PMC7112131

